# Assessment of health professionals and patients perceived quality of a newly established Emergency Department

**DOI:** 10.1186/1757-7241-20-S2-O2

**Published:** 2012-04-16

**Authors:** Maria Søe Mattsson, Hanne Jørsboe

**Affiliations:** 1 Emergency Department, Hospital of Nykøbing Falster, Denmark

## Background

The objective of the establishment of the Emergency Departments (ED) in Denmark is to optimize patient treatment. The aim of this study was to explore if the efforts in the ED have an effect on how patients perceive quality. We looked at pathways in acute care and analyzed if there was a relationship between the establishment of the ED and patient perceived quality with a special focus on medication and information.

## Methods

The study was based on a questionnaire survey and compared to an audit study concerning the same patient group. The questionnaire contained 15 questions. The study was initiated in 2009 and lasted 12 weeks. All adult acute patients facing discharge and who had stayed for a minimum of two hours in the department were included. The audit study was performed using a fixed set of indicators, based on a Danish Quality Model. Chi-square (χ^ 2^) was used for significance testing.

## Results

383 patients participated in the study (193 men, 190 women, mean age 64 years). The response rate was 64 %. The 36 % who did not answer were either too ill to participate in the interview or too quickly discharged.

92 % of the patients answered that they were satisfied with the treatment. 86 % experienced that they received the information they needed during their hospitalization. The audit showed that informed consent concerning treatment was signed in 53 % of the cases.

During their stay 1.8% of the patients experienced errors concerning medication. The audit showed that 64% of the patients had their medication records looked through and signed by a medical doctor.

## Conclusion

We found a correlation between patients' concerning satisfaction with the EDs treatment and different background variables, like gender and age (p <0.05).

There was no correlation between whether patients felt they received the information they needed and the information and informed consent concerning treatment was documented for the medical records.

Furthermore, there was no correlation between the patients’ experience of errors in receiving medication and the number of patients having their medication records signed by a medical doctor. A follow-up survey will be conducted fall 2012.

